# How to assign a (3 + 1)-dimensional superspace group to an incommensurately modulated biological macromolecular crystal

**DOI:** 10.1107/S1600576717007294

**Published:** 2017-06-30

**Authors:** Jason Porta, Jeff Lovelace, Gloria E. O. Borgstahl

**Affiliations:** a The Eppley Institute for Research in Cancer and Allied Diseases, University of Nebraska Medical Center, 986805 Nebraska Medical Center, Omaha, NE 68198, USA; b Department of Biochemistry and Molecular Biology, University of Nebraska Medical Center, 985870 Nebraska Medical Center, Omaha, NE 68198, USA

**Keywords:** protein X-ray crystallography, aperiodic crystals, incommensurate modulation, superspace groups

## Abstract

Periodic crystal diffraction is described using a three-dimensional (3D) unit cell and 3D space-group symmetry. Incommensurately modulated crystals are a subset of aperiodic crystals that need four to six dimensions to describe the observed diffraction pattern, and they have characteristic satellite reflections that are offset from the main reflections. These satellites have a non-integral relationship to the primary lattice and require **q** vectors for processing. Incommensurately modulated biological macromolecular crystals have been frequently observed but so far have not been solved. The authors of this article have been spearheading an initiative to determine this type of crystal structure. The first step toward structure solution is to collect the diffraction data making sure that the satellite reflections are well separated from the main reflections. Once collected they can be integrated and then scaled with appropriate software. Then the assignment of the superspace group is needed. The most common form of modulation is in only one extra direction and can be described with a (3 + 1)D superspace group. The (3 + 1)D superspace groups for chemical crystallographers are fully described in Volume C of *International Tables for Crystallography*. This text includes all types of crystallographic symmetry elements found in small-molecule crystals and can be difficult for structural biologists to understand and apply to their crystals. This article provides an explanation for structural biologists that includes only the subset of biological symmetry elements and demonstrates the application to a real-life example of an incommensurately modulated protein crystal.

## Abstract 2

(3 + 1)-dimensional superspace groups are explained for incommensurately modulated macromolecular crystals with an example.

## 1. Introduction   

Our laboratory is interested in solving the structures of incommensurately modulated protein crystals. These crystals have a fascinating diffraction pattern with satellite reflections surrounding the main reflections. Commensurate and incommensurate macromolecular crystallography, with examples of such effects, as well as twinning and multiple crystal cases were reviewed by Helliwell (2008 ▸) and are also discussed in Chapter 8 of Rupp’s *Biomolecular Crystallography* text book (Rupp, 2010 ▸). This paper concerns our symmetry analysis of the diffraction from (3 + 1)-dimensionally incommensurately modulated crystals of profilin:actin (PA) (Lovelace *et al.*, 2008 ▸; Porta *et al.*, 2011 ▸). This publication relies heavily on our study of an article by van Smaalen (2005 ▸), Chapters 1, 2 and 3 of van Smaalen’s textbook on *Incommensurate Crystallography* (van Smaalen, 2007 ▸) and *International Tables for Crystallography*, Volume C, Chapter 9.8, *Incommensurate and Commensurate Modulated Structures*, by Janssen *et al.* (1999 ▸). We also studied Schönleber’s lectures on *Introduction to Superspace Symmetry* from the Workshop on Structural Analysis of Aperiodic Crystals held in Bayreuth, Germany, and an article by Wagner & Schönleber (2009 ▸). Although these are excellent sources, they were written for small-molecule crystallographers and physicists and use language and examples that are not encountered in macromolecular crystallography. Therefore, we decided to write this paper for the next biological crystallographer who chooses to solve a modulated crystal, so that it will not be so difficult for them to understand and to confidently assign their superspace group to the crystal diffraction data.

In this article the nomenclature common to periodic three-dimensional (3D) crystals is used with adaptations to a fourth dimension as needed (Janssen *et al.*, 1999 ▸). It is noteworthy that in much of the aperiodic literature another formalism is used, where subscripts *i* = 1, 2, 3 are used to indicate the space directions (van Smaalen, 2007 ▸). This makes it easier to add more dimensions as needed. Hence, the symbols (**a**, **b**, **c**), (*x*, *y*, *z*), (*hkl*) and (α, β, γ) used in this publication correspond to (**a**_1_, **a**_2_, **a**_3_), (*x*_1_, *x*_2_, *x*_3_), (*h*_1_ *h*_2_ *h*_3_) and (σ_1_, σ_2_, σ_3_), respectively, in aperiodic crystallography. Vectors are in bold and scalar coefficients are in italics. This is pointed out here to help avoid confusion when reading the aperiodic literature.

**Figure 1:**
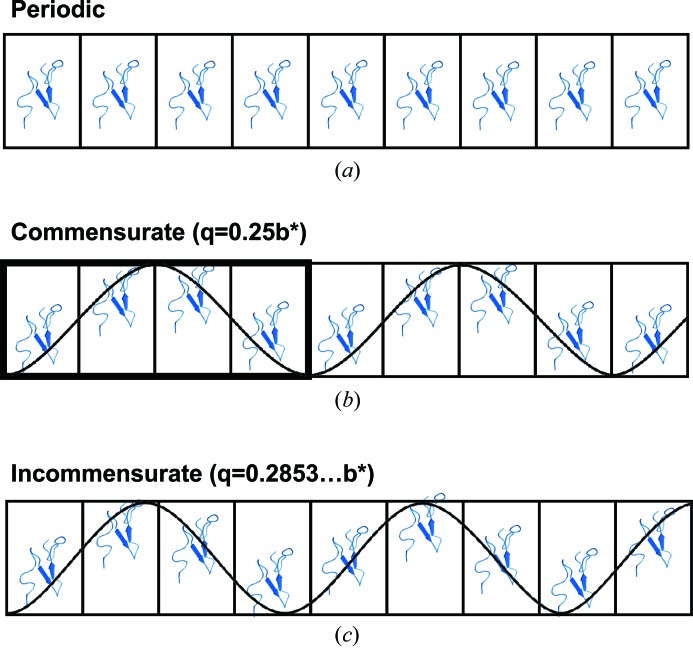
Three categories of crystals. (*a*) Periodic case with identical unit cells. (*b*) Commensurate modulation. (*c*) Incommensurate modulation with harmonic modulation wave. Protein structure from PDB entry 2rro. This figure is reproduced from Porta *et al.* (2011 ▸). The modulation here is kept simple for the purposes of instruction. Modulations can be complex and involve translations, rotations, variations in occupancies, subdomains and/or a combination.

Crystal periodicities can be categorized into three types (Fig. 1 ▸). The first is the most common case, where the crystal is periodic and the unit-cell contents are duplicated closely by the lattice translations (Fig. 1 ▸ *a*). The second type is the case of a commensurate modulation. Here, the spacing of the satellite reflections relative to the main reflections is a rational value. The diffraction pattern can be indexed and integrated with any protein crystallography data reduction software with three integer indices as a supercell. In Fig. 1 ▸(*b*), the **q** vector which is used in aperiodic crystallography to index the satellite reflections relative to the main reflection has a rational value of 0.25 (or 1/4) and the modulation of the structure repeats every four unit cells. This means that the lattice parameters for indexing the satellite reflections are integer multiples (1, 2,…, *n*) and the crystal structure can be described with a supercell (Wagner & Schönleber, 2009 ▸). The third type is the case of an incommensurately modulated crystal. Here, at least one component of the **q** vector is irrational and cannot be calculated with a simple fraction (Fig. 1 ▸ *c*). An accurate description of an incommensurately modulated crystal can only be obtained by describing the diffraction pattern with **q** vectors.

When an incommensurately modulated diffraction pattern is observed in protein crystallography, the sample is typically discarded in favour of a better behaving sample that can be processed with standard macromolecular crystallography software. As a consequence, incommensurately modulated macromolecular crystals are rarely reported and these types of structural modulations in the context of a macromolecular crystal are poorly understood. PA crystals can be chemically induced to form a peculiar incommensurately modulated diffraction pattern. More than 28 years ago (Schutt *et al.*, 1989 ▸) it was found that when PA crystals are driven to a phase transition boundary by exposing the crystals to conditions known to promote actin filament formation they transform into an incommensurately modulated state that is thought to contain a superstructure of structural intermediates. By varying the solution conditions PA can be crystallized in either an ‘open’ or a ‘closed/tight’ state that corresponds to the nucleotide binding site opening and closing (Chik *et al.*, 1996 ▸; Porta, 2011 ▸; Schutt *et al.*, 1993 ▸). These two states are accompanied by a change in the *c* unit-cell dimension from 186 to 172 Å, respectively. Incommensurate diffraction was obtained using precession photography at room temperature from either open or closed states by shifting the pH to 6.0, a condition known to cause profilin to diffuse away from actin and actin filaments to form *in vitro* (Carlsson, 1979 ▸; Oda *et al.*, 2001 ▸; Chik, 1996 ▸). This research provided the foundation for our continued studies.

**Figure 2:**
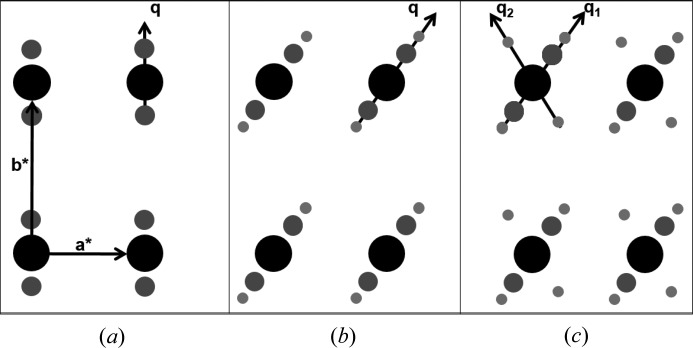
(3 + *d*)D incommensurate diffraction scenarios, where *d* = 1, 2, or 3. (*a*) (3 + 1)D modulation with a single **q** vector parallel to b* and first-order satellites (*m* = ±1). (*b*) (3 + 1)D with second-order satellites (*m* = ±2) and a single **q** vector in the *ab* plane. (*c*) (3 + 2)D diffraction with two **q** vectors. The large black circles represent the ‘main’ reflections and the smaller grey and light-grey circles represent the ‘satellite’ reflections. This figure was reproduced from Porta *et al.* (2011 ▸).

Incommensurate modulations within crystals are a result of a displacement modulation that forms but does not align with the spacing of the basic unit cell. In the resulting diffraction pattern satellite reflections appear near the normal main reflections (see Fig. 2 ▸). In the periodic state, all reflections can be indexed by the three integer indices *h*, *k* and *l* such that
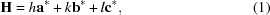
where a*, b* and c* are the reciprocal lattice vectors of the main reflections and basic unit cell. With satellite reflections, the diffraction pattern becomes (3 + *d*) dimensional, where *d* is the number of satellite directions. The most common form of modulation is in only one extra direction (*d* = 1), and the diffraction patterns for these crystals have satellite reflections on either side of the main reflection (see Figs. 2 ▸ *a* and 2 ▸ *b*). This is called a (3 + 1)D modulated crystal. The diffraction pattern for this case can be indexed by the introduction of a single **q** vector such that
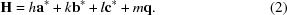
The positions of the satellite reflections are given by the **q** vector
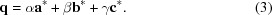
A modulation wave can be parallel to one of the reciprocal lattice vectors, and in this case two of the scalar **q** coefficients in (3) would be zero. In more complicated cases two or three of the **q** coefficients can be nonzero (Fig. 2 ▸ *b*). Also, multiple-order satellites evenly spaced from the main reflections can exist (see Figs. 2 ▸ *b* and 2 ▸ *c*). This is represented by the integer value *m* in equation (2). Interestingly satellites and multi-order satellites are predominantly in the high-resolution bins of data (see Fig. 5 of Lovelace *et al.*, 2010 ▸). In 2008, the Borgstahl laboratory was able to reproduced the incommensurately modulated PA crystals from the Schutt laboratory and measured a single-rotation-style diffraction image from a room-temperature protein crystal (Lovelace *et al.*, 2008 ▸). The data were indexed and the first **q** vector was measured for a macromolecular crystal. Research progress was hindered by the reversibility of the modulation at room temperature, perhaps due to crystal heating or radiation damage from the SuperBright FRE X-ray generator, which prevented the collection of a full set of diffraction data. We have since learned to cryocool crystals that were first crosslinked with acidic glutaraldehyde at room temperature and then cryopreserved with sodium formate (named xMod1 and xMod2, Table 1 ▸) and more recently not crosslinked and cryopreserved with d-glucose (gMod3, Table 1 ▸).

All of the (3 + *d*)D superspace groups have been tabulated for *d* = 1, 2 or 3. For *d* = 1 there are 775 groups, for *d* = 2 there are 3338 groups and for *d* = 3 there are 12 584 groups (Stokes *et al.*, 2011 ▸). A web site has been developed for searching all 775 (3 + 1)D superspace groups listed in *International Tables for Crystallography* (http://it.iucr.org/resources/finder/; Orlov *et al.*, 2008 ▸). These numbers are greatly reduced for biological crystals as there are only 65 chiral (or biological) three-dimensional space groups. Then there are only 135 (3 + 1)D, 368 (3 + 2)D and 1019 (3 + 3)D chiral superspace groups (van Smaalen *et al.*, 2013 ▸). An excellent primer to the three-dimensional space groups was written by Dauter & Jaskolski (2010 ▸) and can be used to review the symmetry elements found in protein crystals (Dauter & Jaskolski, 2010 ▸). Modulated PA crystals have a (3 + 1)D-type superspace group because they are modulated in only one direction.

**Figure 3:**
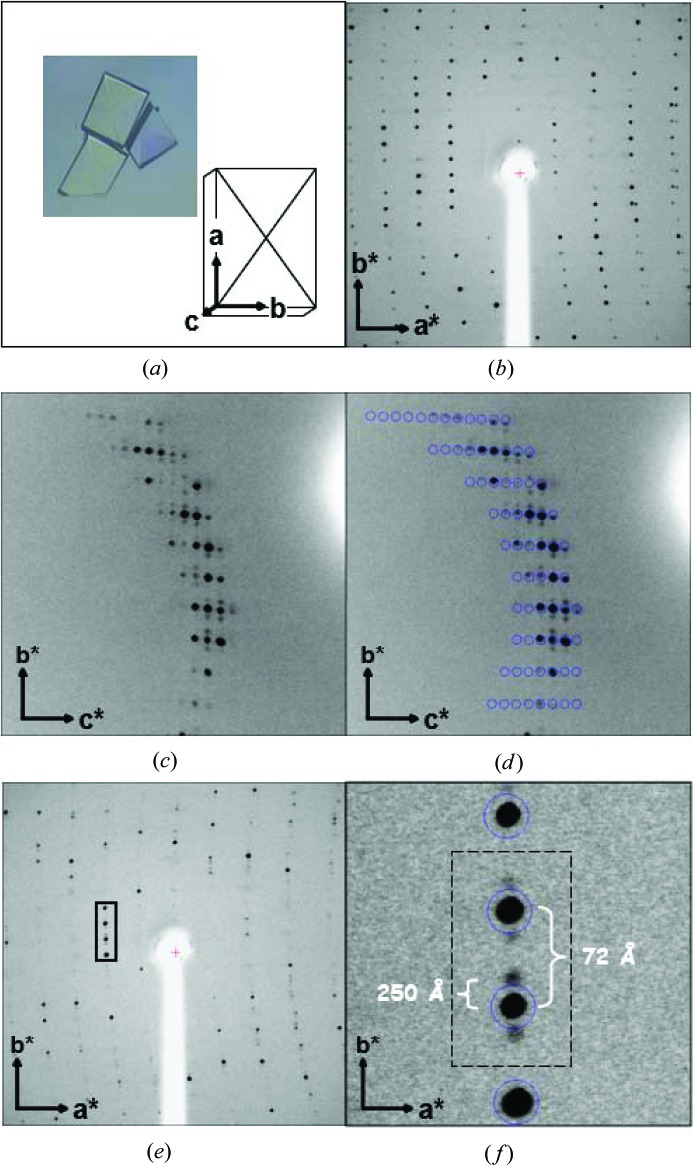
Profilin:actin crystals and diffraction patterns. (*a*) Typical profilin:actin crystal with a diagonal cross along the 001 plane. (*b*) Normal periodic diffraction pattern (Porta & Borgstahl, 2012 ▸). (*c*) Incommensurately modulated diffraction pattern from crystal gMod3 with satellite reflections along b*. (*d*) Same as part (*c*) but with the main reflections circled in blue from the predicted positions calculated by the *CrystalClear* software (Rigaku, Tokyo, Japan). Note the satellite reflections are not predicted; only the main reflections are predicted. (*e*) Incommensurately modulated diffraction pattern from crystal xMod1 with satellite reflections in the b* direction. (*f*) Close-up of the modulated diffraction with approximated unit-cell dimensions. The blue circles are predicted reflection positions. Again, the satellite reflections are not predicted; only the main reflections are predicted. Images were collected at a distance of 300 mm with 15 min exposures and an oscillation of Δφ = 0.5°. Parts (*a*)–(*c*), (*e*) and (*f*) of this figure were reproduced from Lovelace *et al.* (2008 ▸). Note that the crystal diffraction data were processed with *Eval15*, not the *CrystalClear* software. *CrystalClear* was used here for the purposes of illustration only.

The three cryocooled modulated PA data sets (Table 1 ▸) all have basic three-dimensional unit cells like that of the PA open-state crystals and have satellite reflections along b* (Fig. 3 ▸). The crystals differ in their resolution of diffraction and the extent of modulation, as indicated by their **q** vector and satellite intensity strength. The *q* spacing of the satellite from the main reflections varies from 0.2628 to 0.2829. A demonstration of their similarity to and differences from each other and from open-state crystals was made by calculating *R*_merge_ between data sets (Table 2 ▸). The easiest *R*_merge_ to calculate employs the main reflections only. This statistic demonstrates that the crystals are not isomorphous with each other. When the satellite intensities are added to the main reflections the *R*_merge_ values improve and fall in a range of 25–37%, still not isomorphous. Clearly the three modulated structures are significantly different from each other and from the periodic crystal. The intensity of the modulation is also indicated by the strength of the satellite intensities (*e.g.* in Table 1 ▸ *I*/σ for the satellite reflections ranks their strength as follows: gMod3 > xMod2 = xMod1).

**Figure 4:**
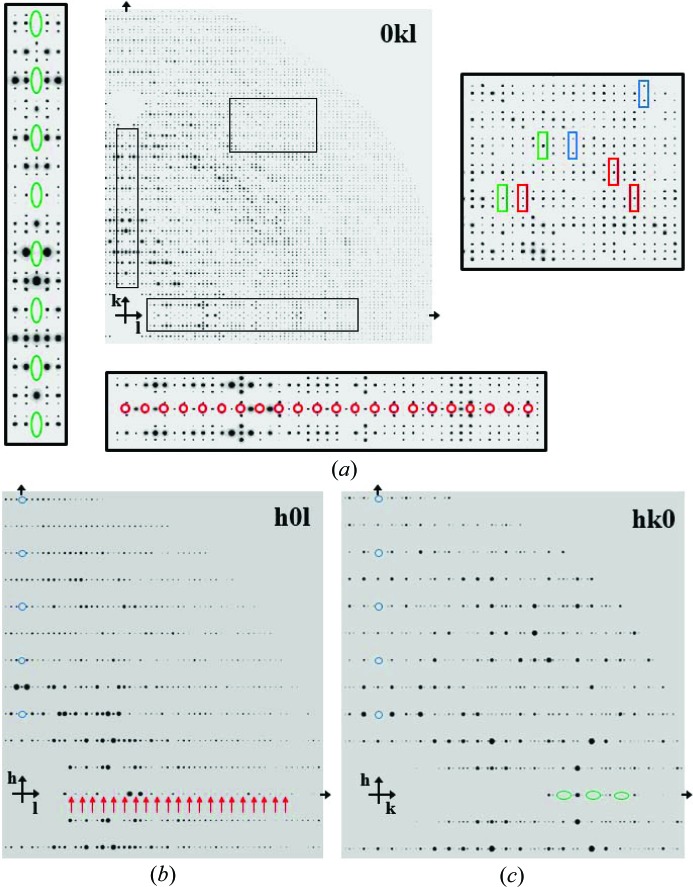
Pseudo-precession photographs of diffraction data from the gMod3 crystal (see Table 1 ▸). Diffraction data, main reflections and satellites, were integrated using *Eval15* as described previously (Porta *et al.*, 2011 ▸). In part (*a*) the 0*kl* plane is displayed with portions of the *k* axis, *l* axis and centre portion magnified. Systematic absences along *k* are highlighted with green ovals and along *l* with red circles. In the centre portion zoom, red rectangles highlight reflections where the satellites do not have equal intensity, green rectangles show where there is just a main reflection with no satellites, and blue rectangles show examples where the satellites extinguish the main reflection. Part (*b*) shows the *h*0*l* plane and part (*c*) the *hk*0 plane, with systematic absences along *h* circled in blue, along *l* highlighted with red arrows and along *k* with green ovals. To prepare these pseudo-precession photographs, the reflections were reindexed to a supercell using an Awk script to reindex the *k* reflections under the supercell condition *k* = 7*k* + 2*m*, where *m* is the satellite order (*m* = ±1 in this study). Reindexing the data into a supercell is illustrated in Fig. S1 of the supporting information. The reindexed reflections were converted to realistic pseudo-precession photographs with the *MLFSOM* software, which applies a point-spread function (Holton, 2008 ▸; Holton *et al.*, 2012 ▸).

We have three cryocooled incommensurately modulated PA structures to solve of varying modulation strength (Fig. 3 ▸). When we look at the gMod3 crystal more closely in pseudo-precession photographs, it can be seen how the satellites relate to the main reflections (Fig. 4 ▸). Satellites are not always present (green rectangles, Fig. 4 ▸ *a*), do not have to be of equal intensity (red rectangles, Fig. 4 ▸ *a*) and can extinguish the main reflections (blue rectangles, Fig. 4 ▸ *a*). The relative intensities between the satellite and the main reflections are analysed by resolution bin in Table 3 ▸ for the gMod3 crystal (see also Fig. 7 and Table 2 of Porta *et al.*, 2011 ▸). The ratio of the satellite to main reflection intensity is lower in low-resolution bins and increases in the high-resolution bins. This is a general feature of modulated PA crystals.

## 2. Assignment of a superspace group to a protein crystal   

A general procedure for the assignment of a superspace group is given by Janssen *et al.* (1999 ▸). These steps are analysed here with our PA diffraction data and the description of the process is streamlined to include only the symmetry elements found in chiral molecule crystals. Hopefully this example will make these methods more accessible to protein crystallographers.

### 2.1. Determine the Laue class and crystallographic point group   

The Laue group of the diffraction pattern is the point group in three dimensions that transforms every diffraction peak into a peak of the same intensity (except for deviations from Friedel’s law caused by dispersion) (Rupp, 2010 ▸). For biological crystals there are 11 Laue symmetry classes and 11 chiral crystallographic point groups (32 point groups for small molecular crystals). These are triclinic 1, monoclinic 2, orthorhombic 222, tetragonal 4 or 422, trigonal 3 or 32, hexagonal 6 or 622, and cubic 23 or 432 (Table S1 in the supporting information). Processing of the main diffraction data with D*TREK (Table 4 ▸) or with *Eval15* (Table 5 ▸) shows that the Laue class is *mmm* and the point group is 222 (Pflugrath, 1999 ▸; Schreurs *et al.*, 2010 ▸). There are only 23 (3 + 1)D superspace groups with this symmetry.

### 2.2. Find the basic unit cell for the main reflections and a modulation wavevector   

The main reflections are separated from the satellites, usually by intensity, and indexed. Reflection extinctions are used to select the Bravais class for the main reflections (Fig. S2). Note that only noncubic classes are possible for (3 + 1)D modulations because a one-dimensional incommensurate modulation is incompatible with cubic symmetry. The satellites are usually assigned to the main reflections (can be extinct) that they are closest to. Then the direction and dimensions of the **q** vector are determined by fitting the satellites. If possible, it is preferable to place the **q** vector along a reciprocal lattice vector.

PA crystals are of the primitive orthorhombic Bravais lattice. This can be seen in the analysis of just the main reflections (Table 4 ▸, solution 11). Centring-type *P* ortho­rhombic has a low least-squares residual almost as low as *P* triclinic or *P* monoclinic. *C* centring is ruled out by the large least-squares residual. *Eval15* processing also selects primitive orthorhombic as the Bravais lattice (Table 5 ▸). This narrows the assignment down to 15 superspace groups. *Eval15* was used to define the **q** vector, which is in the direction of b*, for each crystal (Porta *et al.*, 2011 ▸). We note that the magnitude of the **q** vector for xMod1 is close to 2/7 = 0.2857… and so 2/7 was used as an approximation when the diffraction was reindexed for display as a pseudo-precession in Fig. 4 ▸ (see also Fig. S1).

### 2.3. Determine the 3D space group of the average structure   

The average structure is commonly found by using the main reflections only and corresponds to averaging the contents of several unit cells in three dimensions. The space group of the average structure is determined from the main reflections. This helps make a good choice for the starting structure in superspace refinement. Tables 4 ▸ and 5 ▸ show that the three-dimensional space group is *P*222. The three-dimensional space group for the average structure is determined from the main reflections. In our case, checks for centring rule out *C*, *F* or *I* and the lattice is primitive. Extinctions along *h*, *k* and *l* (Fig. 4 ▸) indicate the presence of screw axes along all three dimensions. Since the data are of fairly low resolution the assignment of the space group was checked by performing molecular replacement with just the main reflections using *MOLREP* (Table 6 ▸) (Vagin & Teplyakov, 2000 ▸). This settles any uncertainty and the 3D space group of the average structure is *P*2_1_2_1_2_1_.

**Figure 5:**
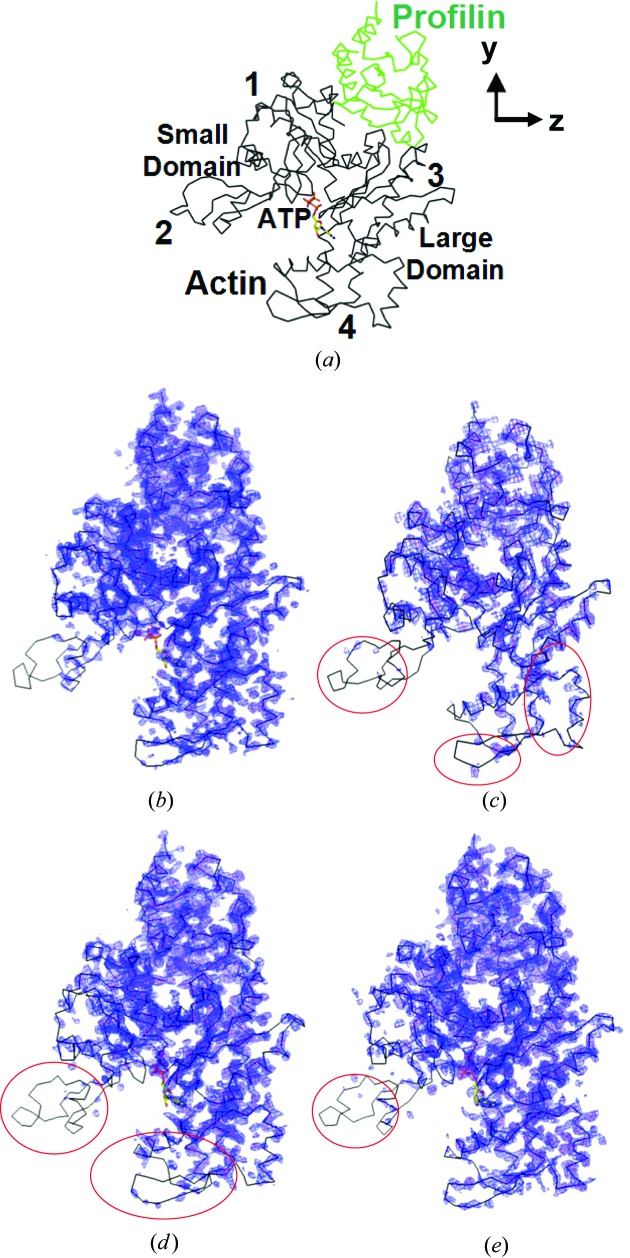
Analysis of the average structure in modulated PA crystals. (*a*) Cα trace of profilin:actin with the domains of actin labelled. (*b*)–(*e*) 2*F*_o_–*F*_c_ electron density (2σ, blue; 3σ, red) of (*b*) wide-open state, periodic structure at 2.3 Å resolution, and (*c*) xMod1, (*d*) xMod2 and (*e*) gMod3 average modulated structures (main reflections + satellites merged with *JANA2006*). Regions in the average maps with density that is weaker than in the periodic map (circled in red) are the locations in the structure with multiple positions owing to modulation.

At this point a refinement of the average structure can be performed and the resulting electron density observed. The average structures refined with *REFMAC* crystallographic *R* values of 27–28% (Murshudov *et al.*, 1997 ▸). The electron density of the average structure (Fig. 5 ▸) reveals that some parts of the structure are modulated more than others. The average electron density for profilin and subdomains 1 and 3 of actin are fairly well ordered. Actin domains 2 and 4 have very weak density, and this indicates that their motions are more dramatic in the modulation wave. The modulation function in actin appears to be more pronounced than that in profilin.

**Figure 6:**
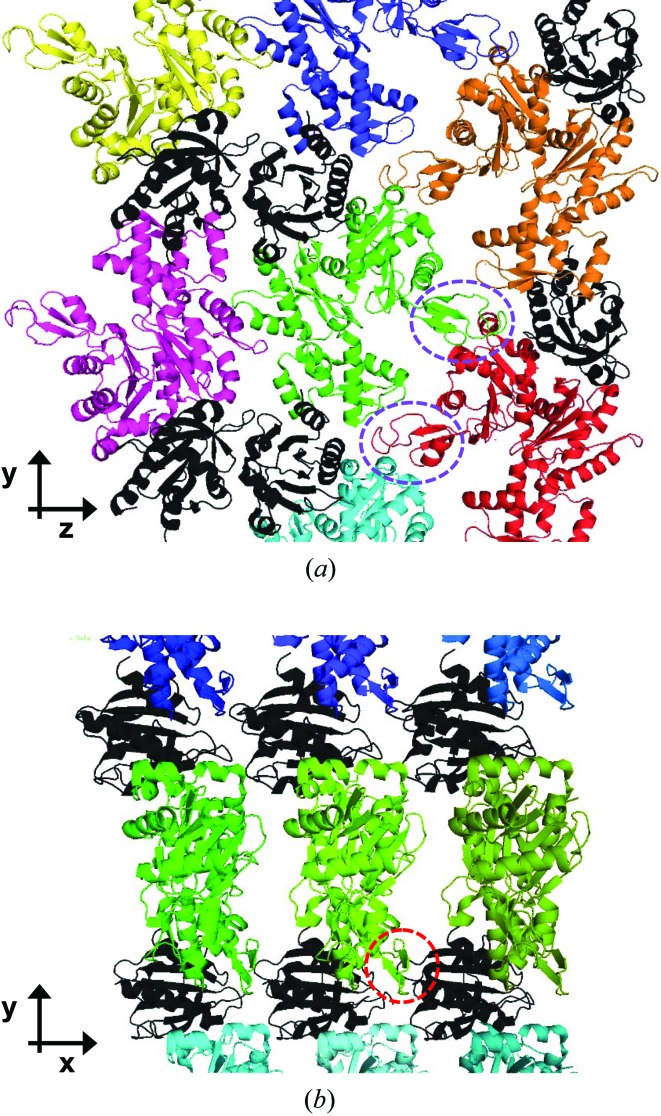
The crystal lattice of profilin:actin crystals (*a*) looking down **x** and (*b*) looking down **z**. In both views the direction of the actin ribbon formed in these crystals is along **y** and vertical. In (*a*) one complete actin ribbon is seen on the right half of the figure, composed of four profilins (black) and five actins (cyan, red, green, orange and blue). Domain 2 in the actin small domain is indicated with a dashed circle in magenta in part (*a*) and red in part (*b*).

An illustration of the crystal contacts in PA crystals shows how the modulated regions correspond to the crystal directions (Fig. 6 ▸). For the PA case, the indexing showed that the modulation is along **b** (collinear with **y**) in the crystal, which corresponds to an ‘actin ribbon’ formed by the crystal lattice (Schutt *et al.*, 1991 ▸). It is likely that the protein undergoes a conformational change that affects the neighbouring PA molecules in such a way as to produce the observed modulation in the diffraction pattern (Schutt *et al.*, 1991 ▸). The structural basis for the modulated PA diffraction pattern has not yet been determined.

### 2.4. Identification of the (3 + 1)D Bravais lattice type   

The (3 + 1)D Bravais class is determined by the 3D Bravais class and the components α, β, γ of **q**. Next we find the superspace group compatible with the previously derived results and with the special extinctions observed in the diffraction pattern. In Table S2 there are 15 orthorhombic (3 + 1)D superspace groups (Nos. 16.1–19.1). From the main reflections we know our lattice is primitive and there is no centring. There are three screw axes. Applying the screw axes narrows the selection down to one and the superspace group for the incommensurately modulated PA crystals is number 19.1 with notation *P*2_1_2_1_2_1_(00γ). There are actually three related versions of this space group: *P*2_1_2_1_2_1_(α00), *P*2_1_2_1_2_1_(0β0) and *P*2_1_2_1_2_1_(00γ). The direction of the modulation is shown by the position of the coefficients. For PA the orientation is *P*2_1_2_1_2_1_(0β0). The symmetry operators need to be modified to work with modulation along this axis relative to c* as reported in the tables. Details of this transformation were reported by Lovelace *et al.* (2013 ▸).

## 3. Conclusions   

After integration of reflections in *Eval15* the unit-cell dimensions and **q** vector length and direction are known. The final test of the superspace group assignment comes in the next step when it is applied to the integrated diffraction data *via* the *SADABS* software (Sheldrick, 1996 ▸). The program workflow is presented in Fig. 3 of Porta *et al.* (2011 ▸). It can be seen in Table 1 ▸ that the *R*_sym_ values obtained from *SADABS* look reasonable and are quite good for the well measured data of <4 Å resolution.

During data collection it was noticed that particularly strong satellite reflections were associated with extinguished main reflections (Fig. 4 ▸). As it turns out, this is indicative of large movements in the structural modulation (Janssen *et al.*, 1999 ▸). It is interesting to note that, when normal periodic actin in PA crystals undergoes a transition from the ‘open’ to ‘tight’ state, the unit-cell dimension *c* changes by 14 Å, yet the crystals are stable (Chik, 1996 ▸). It is therefore possible that the structural transitions needed to bring about such a large modulation might be on a similar scale, especially those involving actin subdomains 1 and 4. Refinement of the incommensurate PA structures will inevitably shed light on the nature of these higher-order actin structures and provide insight into the early stages of actin filament formation. This is the next step in our research and involves further software development for crystallographic refinement of a protein in a (3 + 1)D superspace group.

## Supplementary Material

Supporting information file. DOI: 10.1107/S1600576717007294/gj5178sup1.pdf

## Figures and Tables

**Table 1:** *Eval15* data processing statistics for incommensurately modulated PA diffraction data Data for the highest-resolution shell are in parentheses.

Crystal name	xMod1	xMod2	gMod3
Crystal treatment†	Crosslinked 25% glutaraldehyde in 0.001 *N* HCl, 7 *M* sodium formate	Crosslinked 25% glutaraldehyde in 0.001 *N* HCl, 7 *M* sodium formate	Soaked in 70%(*w*/*v*) D-glucose
Beamline	Rigaku FRE/VariMaxHR	NSLS X4C	Rigaku FRE/VariMaxHR
Temperature (K)	100	100	100
Maximum resolution (Å)	3.0	2.5	2.4
Completeness (%)	98.2 (85.9)	96.3 (95.5)	99.9 (100)
Unit-cell parameters			
*a* (Å)	37.3	37.6	38.0
*b* (Å)	71.1	71.1	71.9
*c* (Å)	185.1	186	186.9
α, β, γ (°)	90	90	90
**q** vector, order	0a* + 0.2829b* + 0c*, *d* = 1	0a* + 0.2628b* + 0c*, *d* = 1	0a* + 0.2700b* + 0c*, *d* = 1
〈*I*/σ〉 [main; satellites]	[15.2 (4.2); 2.2 (1.4)]	[3.4 (1.6); 1.9 (0.73)]	[7.5 (2.2); 7.6 (1.8)]
〈*I*/σ〉 [main; satellites] 23–4.0 Å	[15.9 (10.8); 3.1 (2.2)]	[4.8 (4.8); 2.9 (3.1)]	[14.6 (14.5); 15.8 (15.9)]
No. unique reflections [main; satellites]	[10 669; 21 532]	[20 541; 39 852]	[23 631; 42 530]
Laue group	222	222	222
*R*_sym_ (%)‡	8.2 (56.3)	11.4 (33.4)	10.6 (37.3)
*R*_sym_ (%) 23–4.0 Å	7.3 (30.3)	8.3 (10)	6.9 (8.1)

† Wide-open-state profilin:actin crystals were grown and then treated. The open-state unit-cell dimensions *a*, *b* and *c* are 38, 72 and 186.8 Å, respectively (Porta & Borgstahl, 2012 ▸).

‡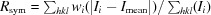.

**Table:** Calculated with main reflections only.

	Periodic	xMod1	xMod2	gMod3
Periodic	0	0.371	0.616	0.307
xMod1		0	0.665	0.284
xMod2			0	0.738
gMod3				0

**Table:** Calculated with main reflection intensities added to satellites.

	Periodic	xMod1	xMod2	gMod3
Periodic	0	0.362	0.372	0.292
xMod1		0	0.281	0.281
xMod2			0	0.255
gMod3				0

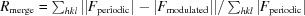
. *R*_merge_ < 0.1 means the datasets are very isomorphous. If *R*_merge_ > 0.3 there is a lack of isomorphism between the crystals. The periodic crystallographic data were reported by Porta & Borgstahl (2012 ▸) as the ‘wide-open-state’ crystal.

**Table 3:** Statistics on the relative intensity of satellite-to-main reflections for crystal gMod3

Resolution bin (Å)	No. of reflections	Average ratio†	Standard deviation ratio	Maximum ratio	Rejects (ratio > 1)‡
26.88–5.66	8931	0.14	0.19	25.53	715
5.66–4.45	8180	0.22	0.24	27.45	1467
4.45–3.87	7645	0.26	0.25	42.01	2001
3.87–3.49	6941	0.31	0.27	21.77	2705
3.49–3.21	6753	0.31	0.27	12.58	2893
3.21–3.00	6686	0.33	0.28	13.28	2961
3.00–2.82	6874	0.35	0.28	8.20	2772
2.82–2.67	6925	0.38	0.29	19.74	2721
2.67–2.52	7221	0.39	0.29	6.31	2426
2.52–2.36	7443	0.41	0.28	6.02	2202

† Ratio = *I*_satellite_/*I*_main_. Only reflections with strong main reflections [*I*_main_/σ(*I*_main_) > 2.5] were used. The average and standard deviations were calculated from the ratio distribution after reflections with a ratio greater than 1 had been rejected.

‡ Cases where *I*_satellite_ > *I*_main_ were excluded from the calculation. A similar table for the xMod1 crystal was published by Porta *et al.* (2011 ▸).

**Table 4:** Processing of main reflections with d*TREK for crystal xMod1

						*a* (Å)	*b* (Å)	*c* (Å)
Solution number	Least-squares residual (%)	Space-group number	Centring type	Bravais cell type	Volume (Å^3^)	α (°)	β (°)	γ (°)
9	2.868	21	*C*	Orthorhombic	671 047	26.4	138.3	183.8
						90	90	90
11	0.032	16	*P*	Orthorhombic	482 346	37.3	70.4	183.8
						90	90	90
12	2.860	5	*C*	Monoclinic	685 828	368.5	26.4	70.4
						90	90.793	90
13	0.012	3	*P*	Monoclinic	482 346	70.4	37.3	183.8
						90	90.02	90
14	0.000	1	*P*	Triclinic	482 346	37.3	70.4	183.8
						90.021	90.016	90.046

**Table 5:** Data processing with *Eval15* for crystal xMod1

Point group	*R*	*R*_meas_	*R*_pim_	χ^2^	*n*Uni	*nR*_sym_	Bravais cell type
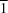	0.134	0.183	0.125	7.95	23912	53619	Triclinic
2/*m*	0.141	0.186	0.118	9.66	25996	71467	Monoclinic
*mmm*	0.119	0.153	0.095	10.81	28097	87773	Orthorhombic
4/*m*	1.124	1.229	0.484	1233.4	17037	85198	Tetragonal low
4/*mmm*	1.207	1.293	0.453	1198.5	12459	88763	Tetragonal high
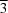	0.838	0.991	0.510	790.3	22881	75520	Trigonal low
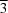 *m*1	1.117	1.249	0.537	1155.4	18061	86616	Trigonal high
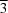 1*m*	1.136	1.255	0.511	1138.3	16767	86629	Trigonal high
6/*m*	0.800	0.887	0.368	701.4	19586	86619	Hexagonal low
6/*mmm*	1.183	1.255	0.404	1051.7	11045	88313	Hexagonal high
*m*3	1.356	1.477	0.568	1144.9	14924	88245	Cubic low
*m*3*m*	1.338	1.390	0.346	757.8	14819	88916	Cubic high

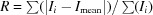
, 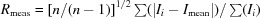, 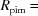 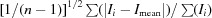, 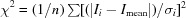, *n*Uni is the number of unique reflections and *nR*_sym_ is the number used to calculate the R values.

**Table 6:** Check of space-group screw axes with *Molrep* with main reflections for crystal xMod1 The score is the product of the correlation coefficient (CC) and packing function [*Q*(*s*)].† The contrast is the ratio of the top score to the mean score.

Space group	Space-group number	Score^‡^	Contrast
*P*222	16	0.101	0
*P*222_1_	17	0.407	6.899
*P*2_1_22	1017	0.100	0
*P*22_1_2	2017	0.086	0
*P*2_1_2_1_2	18	0.107	0
*P*2_1_22_1_	2018	0.430	9.494
*P*22_1_2_1_	3018	0.437	11.505
*P*2_1_2_1_2_1_	19	0.632	18.925

† Score = *Q*(*s*) × CC, where 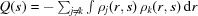 and 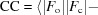 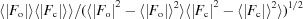.
