# Supplementary material for: How to assign a (3 + 1)-dimensional superspace group to an incommensurately modulated biological macromolecular crystal
Source: J Appl Crystallogr. 2017 Jun 30;50(Pt 4):1200–7. doi: 10.1107/S1600576717007294 (PMC5541356; doi:10.1107/S1600576717007294)
Supplement: Supplementary file 1 [file j-50-01200-sup1.pdf]

JOURNAL OF
APPLIED
CRYSTALLOGRAPHY

Volume 50 (2017)

Supporting information for article:

How to Assign a (3+1)D Superspace Group to an Incommensurately Modulated Biological Macromolecular Crystal

Jason Porta, Jeff Lovelace and Gloria E. O. Borgstahl

Supporting information

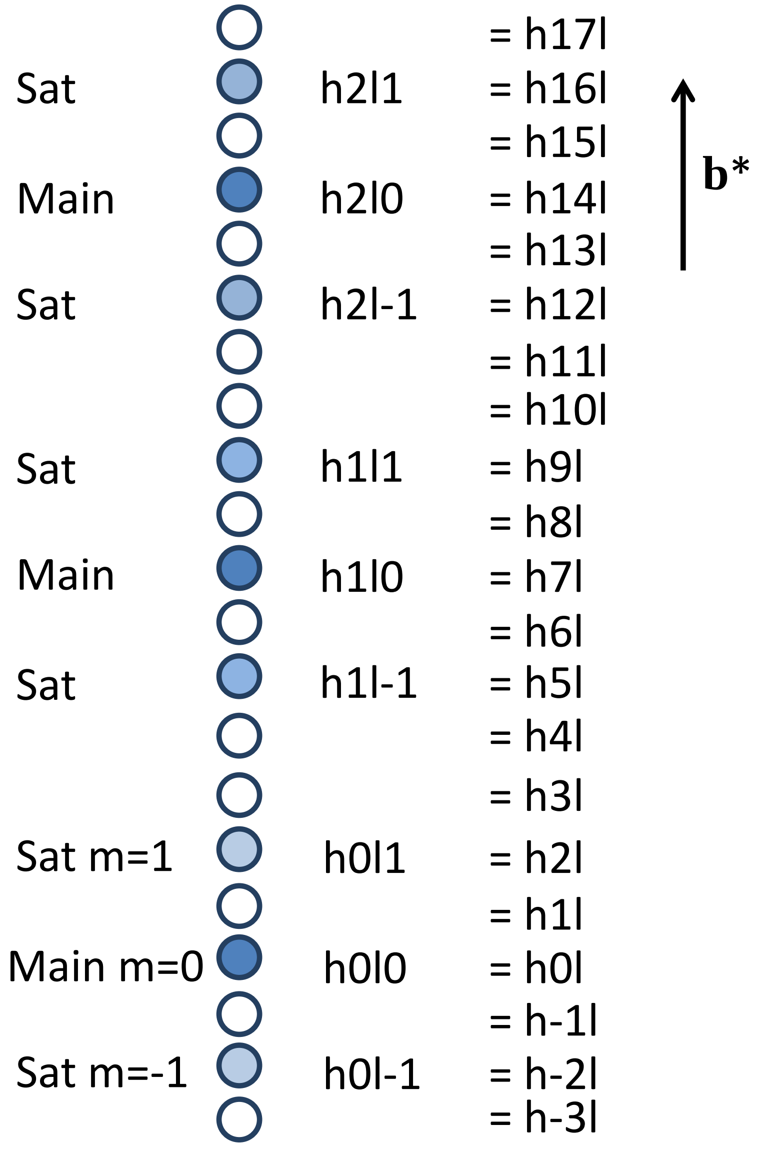

Figure S1

Supercell reindexing of modulated PA data. Reflections coloured dark blue are for main reflections and light blue for satellites. Absent reflections are white. The diagram shows the increasing k index from the bottom to the top. On the right-hand column, the supercell index after applying the condition k = 7k+2m is listed.

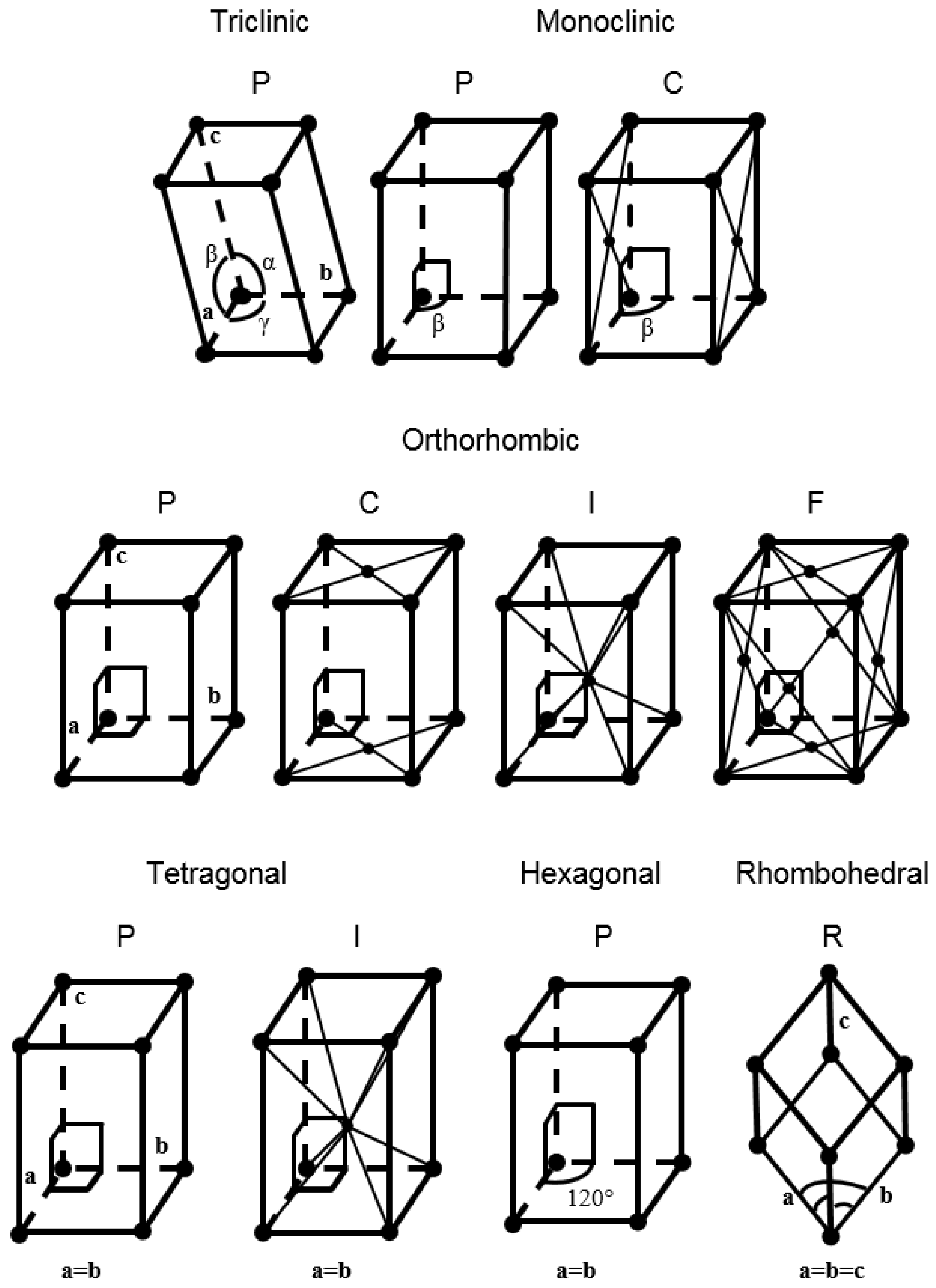

Figure S2 The Bravais lattices possible for (3+1)D incommensurately modulated crystals. Please note the α, β, and γ refer to the unit cell angles (not the q vector coefficients).

Table S1 Laue symmetry classes and chiral point groups

System	Laue Class	Point Group
Triclinic	$\bar{1}$	**1**
Monoclinic	2/m	**2**
Orthorhombic	mmm	**222**
Tetragonal	4/m	**4**
	4/mmm	**422**
Trigonal	$\bar{3}$	**3**
	$\bar{3}$m	**32**
Hexagonal	6/m	**6**
	6/mmm	**622**
Cubic	m$\bar{3}$	**23**
	m$\bar{3}$m	**432**

Table S2

(3+1)-Dimensional superspace groups for incommensurate macromolecular structures. The 135 biological (3+1)D superspace groups are listed here, see Janssen *et al.* (1999) Table 9.8.3.5 for the complete table listing all 775 (3+1)D superspace groups. The number labelling the superspace group is denoted by *n.m*, where *n* is the number attached to the three-dimensional space group and *m* numbers the various superspace groups having the same 3D group. The symbol of the associated 3D space group, the symbol for the four-dimensional point group K_s, the number of the four-dimensional Bravais class to which the superspace group belongs (See Table 9.8.3.2(b) in Janssen *et al.* (1999)), and the superspace-group symbol are also given. The superspace-group symbol is indicated in the short notation and then the values of τ are given for each of the generators in this symbol, unless all these values are zero. Then, instead of writing a number of zeros, one omits them all. Finally, the special reflection conditions due to non-primitive translations are given, for *hklm* if $\mathbf{q}^r = 0$ and for *HKLm* otherwise. The *HKLm* are the indices with respect to a conventional basis **a***, **b***, **c***, $\mathbf{q}^i$. The reflection conditions due to centring translations are given in Table 9.8.3.6 of Janssen *et al.* (1999).

No.	3D space group	Point group K_s	Bravais class No.	Group symbol	Special reflection conditions
1.1	$P1$	(1, 1)	1	$P1(\alpha\beta\gamma)$	
3.1	$P2$	$(2, \bar{1})$	2	$P2(\alpha\beta0)$	
3.2		$(2, \bar{1})$	3	$P2(\alpha\beta\frac{1}{2})$	
3.3		(2, 1)	5	$P2(00\gamma)$	
3.4		(2, 1)	5	$P2(00\gamma)s$	$00lm$: $m = 2n$
3.5		(2, 1)	6	$P2(\frac{1}{2}0\gamma)$	
4.1	$P2_1$	$(2, \bar{1})$	2	$P2_1(\alpha\beta0)$	$00l0$: $l = 2n$
4.2		(2, 1)	5	$P2_1(00\gamma)$	$00lm$: $l = 2n$
4.3		(2, 1)	6	$P2_1(\frac{1}{2}0\gamma)$	$00Lm$: $L = 2n$
5.1	$B2$	$(2, \bar{1})$	4	$B2(\alpha\beta0)$	
5.2		(2, 1)	7	$B2(00\gamma)$	
5.3		(2, 1)	7	$B2(00\gamma)s$	$00lm$: $m = 2n$
5.4		(2, 1)	8	$B2(0\frac{1}{2}\gamma)$	
16.1	$P222$	$(222, \bar{1}\bar{1}1)$	9	$P222(00\gamma)$	
16.2			9	$P222(00\gamma)00s$	$00lm$: $m = 2n$
16.3			10	$P222(0\frac{1}{2}\gamma)$	
16.4			11	$P222(\frac{1}{2}\frac{1}{2}\gamma)$	
17.1	$P222_1$	$(222, \bar{1}\bar{1}1)$	9	$P222_1(00\gamma)$	$00lm$: $l = 2n$
17.2			10	$P222_1(0\frac{1}{2}\gamma)$	$00Lm$: $L = 2n$
17.3			11	$P222_1(\frac{1}{2}\frac{1}{2}\gamma)$	$00Lm$: $L = 2n$
17.4			9	$P2_122(00\gamma)$	$h000$: $h = 2n$

17.5			9	$P2_122(00\gamma)00s$	$h000$: $h = 2n$; $00lm$: $m = 2n$
17.6			10	$P2_122(0\frac{1}{2}\gamma)$	$H000$: $H = 2n$
18.1	$P2_12_12$	$(222, \bar{1}\bar{1}1)$	9	$P2_12_12(00\gamma)$	$h000$: $h = 2n$; $0k00$: $k = 2n$
18.2			9	$P2_12_12(00\gamma)00s$	$h000$: $h = 2n$; $0k00$: $k = 2n$; $00lm$: $m = 2n$
18.3			9	$P2_122_1(00\gamma)$	$h000$: $h = 2n$; $00lm$: $l = 2n$
18.4			10	$P2_122_1(0\frac{1}{2}\gamma)$	$H000$: $H = 2n$; $00Lm$: $L = 2n$
19.1	$P2_12_12_1$	$(222, \bar{1}\bar{1}1)$	9	$P2_12_12_1(00\gamma)$	$h000$: h = 2n; $0k00$: $k = 2n$; $00lm$: $l = 2n$
20.1	$C222_1$	$(222, \bar{1}\bar{1}1)$	13	$C222_1(00\gamma)$	$00lm$: $l = 2n$
20.2			14	$C222_1(10\gamma)$	$00Lm$: $L = 2n$
20.3			15	$A2_122(00\gamma)$	$h000$: $h = 2n$
20.4			15	$A2_122(00\gamma)00s$	$h000$: $h = 2n$; $00lm$: $m = 2n$
21.1	$C222$	$(222, \bar{1}\bar{1}1)$	13	$C222(00\gamma)$	
21.2			13	$C222(00\gamma)00s$	$00lm$: $m = 2n$
21.3			14	$C222(10\gamma)$	
21.4			14	$C222(10\gamma)00s$	$00Lm$: $m = 2n$
21.5			15	$A222(00\gamma)$	
21.6			15	$A222(00\gamma)00s$	$00lm$: $m = 2n$
21.7			16	$A222(\frac{1}{2}0\gamma)$	
22.1	$F222$	$(222, \bar{1}\bar{1}1)$	17	$F222(00\gamma)$	
22.2			17	$F222(00\gamma)00s$	$00lm$: $m = 2n$
22.3			18	$F222(10\gamma)$	
23.1	$I222$	$(222, \bar{1}\bar{1}1)$	12	$I222(00\gamma)$	
23.2			12	$I222(00\gamma)00s$	$00lm$: $m = 2n$
24.1	$I2_12_12_1$	$(222, \bar{1}\bar{1}1)$	12	$I2_12_12_1(00\gamma)$	$h000$: $h = 2n$; $0k00$: $k = 2n$; $00lm$: $l = 2n$
24.2			12	$I2_12_12_1(00\gamma)00s$	$h000$: $h = 2n$; $0k00$: $k = 2n$; $00lm$: $l + m = 2n$
75.1	$P4$	$(4, 1)$	19	$P4(00\gamma)$	
75.2			19	$P4(00\gamma)q$	$00lm$: $m = 4n$
75.3			19	$P4(00\gamma)s$	$00lm$: $m = 2n$
75.4			20	$P4(\frac{1}{2}\frac{1}{2}\gamma)$	
75.5			20	$P4(\frac{1}{2}\frac{1}{2}\gamma)q$	$00Lm$: $m = 4n$
76.1	$P4_1$	$(4, 1)$	19	$P4_1(00\gamma)$	$00lm$: $l = 4n$

76.2			20	$P4_1(\frac{1}{2}\frac{1}{2}\gamma)$	$00Lm$: $L = 4n$
77.1	$P4_2$	(4, 1)	19	$P4_2(00\gamma)$	$00lm$: $l = 2n$
77.2			19	$P4_2(00\gamma)q$	$00lm$: $2l + m = 4n$
77.3			20	$P4_2(\frac{1}{2}\frac{1}{2}\gamma)$	$00Lm$: $L = 2n$
77.4			20	$P4_2(\frac{1}{2}\frac{1}{2}\gamma)q$	$00Lm$: $2L + m = 4n$
78.1	$P4_3$	(4, 1)	19	$P4_3(00\gamma)$	$00lm$: $l = 4n$
78.2			20	$P4_3(\frac{1}{2}\frac{1}{2}\gamma)$	$00Lm$: $L = 4n$
79.1	$I4$	(4, 1)	21	$I4(00\gamma)$	
79.2			21	$I4(00\gamma)q$	$00lm$: $m = 4n$
79.3			21	$I4(00\gamma)s$	$00lm$: $m = 2n$
80.1	$I4_1$	(4, 1)	21	$I4_1(00\gamma)$	$00lm$: $l = 4n$
80.2			21	$I4_1(00\gamma)q$	$00lm$: $l + m = 4n$
89.1	$P422$	$(422, 1\bar{1}\bar{1})$	19	$P422(00\gamma)$	
89.2			19	$P422(00\gamma)q00$	$00lm$: $m = 4n$
89.3			19	$P422(00\gamma)s00$	$00lm$: $m = 2n$
89.4			20	$P422(\frac{1}{2}\frac{1}{2}\gamma)$	
89.5			20	$P422(\frac{1}{2}\frac{1}{2}\gamma)q00$	$00Lm$: $m = 4n$
90.1	$P42_12$	$(422, 1\bar{1}\bar{1})$	19	$P42_12(00\gamma)$	$h000$: $h = 2n$
90.2			19	$P42_12(00\gamma)q00$	$00lm$: $m = 4n$; $h000$: $h = 2n$
90.3			19	$P42_12(00\gamma)s00$	$00lm$: $m = 2n$; $h000$: $h = 2n$
91.1	$P4_122$	$(422, 1\bar{1}\bar{1})$	19	$P4_122(00\gamma)$	$00lm$: $l = 4n$
91.2			20	$P4_122(\frac{1}{2}\frac{1}{2}\gamma)$	$00Lm$: $L = 4n$
92.1	$P4_12_12$	$(422, 1\bar{1}\bar{1})$	19	$P4_12_12(00\gamma)$	$00lm$: $l = 4n$; $h000$: $h = 2n$
93.1	$P4_222$	$(422, 1\bar{1}\bar{1})$	19	$P4_222(00\gamma)$	$00lm$: $l = 2n$
93.2			19	$P4_222(00\gamma)q00$	$00lm$: $2l + m = 4n$
93.3			20	$P4_222(\frac{1}{2}\frac{1}{2}\gamma)$	$00Lm$: $L = 2n$
93.4			20	$P4_222(\frac{1}{2}\frac{1}{2}\gamma)q00$	$00Lm$: $2L + m = 4n$
94.1	$P4_22_12$	$(422, 1\bar{1}\bar{1})$	19	$P4_22_12(00\gamma)$	$00lm$: $l = 2n$; $h000$: $h = 2n$
94.2			19	$P4_22_12(00\gamma)q00$	$00lm$: $2l + m = 4n$; $h000$: $h = 2n$
95.1	$P4_322$	$(422, 1\bar{1}\bar{1})$	19	$P4_322(00\gamma)$	$00lm$: $l = 4n$
95.2			20	$P4_322(\frac{1}{2}\frac{1}{2}\gamma)$	$00Lm$: $L = 4n$
96.1	$P4_32_12$	$(422, 1\bar{1}\bar{1})$	19	$P4_32_12(00\gamma)$	$00lm$: $l = 4n$; $h000$: $h = 2n$
97.1	$I422$	$(422, 1\bar{1}\bar{1})$	21	$I422(00\gamma)$	
97.2			21	$I422(00\gamma)q00$	$00lm$: $m = 4n$
97.3			21	$I422(00\gamma)s00$	$00lm$: $m = 2n$

98.1	$I4_122$	$(422, 1\bar{1}\bar{1})$	21	$I4_122(00\gamma)$	$00lm$: $l = 4n$
98.2			21	$I4_122(00\gamma)q00$	$00lm$: $l + m = 4n$
143.1	$P3$	(3, 1)	23	$P3(\frac{1}{3}\frac{1}{3}\gamma)$	
143.2			24	$P3(00\gamma)$	
143.3			24	$P3(00\gamma)t$	$00lm$: $m = 3n$
144.1	$P3_1$	(3, 1)	23	$P3_1(\frac{1}{3}\frac{1}{3}\gamma)$	$00Lm$: $L = 3n$
144.2			24	$P3_1(00\gamma)$	$00lm$: $l = 3n$
145.1	$P3_2$	(3, 1)	23	$P3_2(\frac{1}{3}\frac{1}{3}\gamma)$	$00Lm$: $L = 3n$
145.2			24	$P3_2(00\gamma)$	$00lm$: $l = 3n$
146.1	$R3$	(3, 1)	22	$R3(00\gamma)$	
146.2			22	$R3(00\gamma)t$	$00lm$: $m = 3n$
149.1	$P312$	$(312, 11\bar{1})$	23	$P312(\frac{1}{3}\frac{1}{3}\gamma)$	
149.2			24	$P312(00\gamma)$	
149.3			24	$P312(00\gamma)t00$	$00lm$: $m = 3n$
150.1	$P321$	$(321, 1\bar{1}1)$	24	$P321(00\gamma)$	
150.2			24	$P321(00\gamma)t00$	$00lm$: $m = 3n$
151.1	$P3_112$	$(312, 11\bar{1})$	23	$P3_112(\frac{1}{3}\frac{1}{3}\gamma)$	$00Lm$: $L = 3n$
151.2			24	$P3_112(00\gamma)$	$00lm$: $l = 3n$
152.1	$P3_121$	$(321, 1\bar{1}1)$	24	$P3_121(00\gamma)$	$00lm$: $l = 3n$
153.1	$P3_212$	$(312, 11\bar{1})$	23	$P3_212(\frac{1}{3}\frac{1}{3}\gamma)$	
153.2			24	$P3_212(00\gamma)$	$00lm$: $l = 3n$
154.1	$P3_221$	$(321, 1\bar{1}1)$	24	$P3_221(00\gamma)$	$00lm$: $l = 3n$
155.1	$R32$	$(32, 1\bar{1})$	22	$R32(00\gamma)$	
155.2			22	$R32(00\gamma)$t0	$00lm$: $m = 3n$
168.1	$P6$	(6, 1)	24	$P6(00\gamma)$	
168.2			24	$P6(00\gamma)h$	$00lm$: $m = 6n$
168.3			24	$P6(00\gamma)t$	$00lm$: $m = 3n$
168.4			24	$P6(00\gamma)s$	$00lm$: $m = 2n$
169.1	$P6_1$	(6, 1)	24	$P6_1(00\gamma)$	$00lm$: $l = 6n$
170.1	$P6_5$	(6, 1)	24	$P6_5(00\gamma)$	$00lm$: $l = 6n$
171.1	$P6_2$	(6, 1)	24	$P6_2(00\gamma)$	$00lm$: $l = 3n$
171.2			24	$P6_2(00\gamma)h$	$00lm$: $2l + m = 6n$
172.1	$P6_4$	(6, 1)	24	$P6_4(00\gamma)$	$00lm$: $l = 3n$
172.2			24	$P6_4(00\gamma)h$	$00lm$: $2l + m = 6n$
173.1	$P6_3$	(6, 1)	24	$P6_3(00\gamma)$	$00lm$: $l = 2n$

173.2			24	$P6_3(00\gamma)h$	$00lm$: $3l + m = 6n$
177.1	$P622$	$(622, 1\bar{1}\bar{1})$	24	$P622(00\gamma)$	
177.2			24	$P622(00\gamma)h00$	$00lm$: $m = 6n$
177.3			24	$P622(00\gamma)t00$	$00lm$: $m = 3n$
177.4			24	$P622(00\gamma)s00$	$00lm$: $m = 2n$
178.1	$P6_122$	$(622, 1\bar{1}\bar{1})$	24	$P6_122(00\gamma)$	$00lm$: $l = 6n$
179.1	$P6_522$	$(622, 1\bar{1}\bar{1})$	24	$P6_522(00\gamma)$	$00lm$: $l = 6n$
180.1	$P6_222$	$(622, 1\bar{1}\bar{1})$	24	$P6_222(00\gamma)$	$00lm$: $l = 3n$
180.2			24	$P6_222(00\gamma)h00$	$00lm$: $2l + m = 6n$
181.1	$P6_422$	$(622, 1\bar{1}\bar{1})$	24	$P6_422(00\gamma)$	$00lm$: $l = 3n$
181.2			24	$P6_422(00\gamma)h00$	$00lm$: $2l + m = 6n$
182.1	$P6_322$	$(622, 1\bar{1}\bar{1})$	24	$P6_322(00\gamma)$	
182.2			24	$P6_322(00\gamma)h00$	$00lm$: $3l + m = 6n$
